# The relationship between muscle thickness and pennation angle is mediated by fascicle length in the muscles of the lower extremities

**DOI:** 10.1038/s41598-024-65100-6

**Published:** 2024-06-27

**Authors:** Saul Martin-Rodriguez, Juan Jose Gonzalez-Henriquez, Juan Carlos Diaz-Conde, Jose A. L. Calbet, Joaquin Sanchis-Moysi

**Affiliations:** 1 Department of Physical Education, University of Las Palmas de Gran Canaria, 35017 Las Palmas de Gran Canaria, Spain; 2 Research Institute of Biomedical and Health Sciences (IUIBS), 35017 Las Palmas de Gran Canaria, Canary Islands Spain; 3 Department of Mathematics, University of Las Palmas de Gran Canaria, Las Palmas de Gran Canaria, Spain; 4 Department of Physical Performance, The Norwegian School of Sport Sciences, Postboks, 4014 Ulleval Stadion, 0806 Oslo, Norway

**Keywords:** Musculoskeletal system, Physiology

## Abstract

Muscle morphological architecture, a crucial determinant of muscle function, has fascinated researchers since the Renaissance. Imaging techniques enable the assessment of parameters such as muscle thickness (MT), pennation angle (PA), and fascicle length (FL), which may vary with growth, sex, and physical activity. Despite known interrelationships, robust mathematical models like causal mediation analysis have not been extensively applied to large population samples. We recruited 109 males and females, measuring knee flexor and extensor, and plantar flexor MT, PA, and FL using real-time ultrasound imaging at rest. A mixed-effects model explored sex, leg (dominant vs. non-dominant), and muscle region differences. Males exhibited greater MT in all muscles (0.1 to 2.1 cm, *p* < 0.01), with no sex differences in FL. Dominant legs showed greater rectus femoris (RF) MT (0.1 cm, *p* = 0.01) and PA (1.5°, *p* = 0.01), while vastus lateralis (VL) had greater FL (1.2 cm, *p* < 0.001) and PA (0.6°, *p* = 0.02). Regional differences were observed in VL, RF, and biceps femoris long head (BFlh). Causal mediation analyses highlighted MT’s influence on PA, mediated by FL. Moderated mediation occurred in BFlh, with FL differences. Gastrocnemius medialis and lateralis exhibited FL-mediated MT and PA relationships. This study unveils the intricate interplay of MT, FL, and PA in muscle architecture.

## Introduction

Structural remodelling of contractile machinery has been a subject of significant research since the pioneering studies of Giovanni Alfonso Borelli and Niels Stensen during the seventeenth century. Their ground-breaking research on the biomechanics of muscles laid the foundation for understanding the intricate relationship between muscle morphology and function, captivating anatomists, and physiologists since the Renaissance^1^. Using imaging techniques^2^ is possible to assess several muscle architecture parameters, including cross-sectional area (CSA), muscle thickness (MT), pennation angle (PA), and fascicle length (FL), which may change with growth, sex, and physical activity. Although it is known that these variables are interrelated, these relationships have yet to be assessed with robust mathematical models and tools like causal mediation analysis (CMA) in ample samples of the population.

During human development, skeletal muscles need to adapt in length as required by the longitudinal bone growth^3^. As a result, skeletal musculature exhibits remarkable adaptability, not only in response to body growth but also to several training stimuli. There is an intricate interplay between MT, FL, and PA which varies not only depending on growth but also on muscle-specific characteristics^4–8^. However, no study has comprehensively explored these relationships in a broad sample of males and females. Understanding the complex interactions among these variables, along with the variability between individuals, could establish a more precise characterization of normal human variability. This would also elucidate the interplay between muscle architecture variables in both sexes.

To address this question, some authors have advocated the use of CMA^9^, a modern statistical approach for understanding the mechanisms by which an exposure or intervention could explain an outcome. This powerful approach has been used in observational research to gain insights into the underlying processes and pathways that contribute to the observed associations in observational data^10^. Mediation can co-occur with moderation, also called conditional indirect effects. In moderated mediation, the influence of a third variable (moderator) on the mediation effect is explored, adding complexity to the relationship analysis^11^. Our laboratory has recently identified through CMA that the FL of the tibialis anterior muscle seems to have a suppressive effect on the PA, suggesting that increments in MT (i.e., set in 10%) are not always aligned with increments in FL or the PA^12^.

In this study, we employed CMA to model the relationship between MT, PA, and FL and investigate how these parameters influence each other using conventional B-mode ultrasonography, the most used technique to assess muscle architecture^2^. By analysing inter and intra-individual heterogeneity in a large sample of human subjects, our research aimed to address two main objectives. Firstly, we sought to determine whether the relationship between the MT on PA is mediated by FL, in a wide range of pennate and non-pennate muscles. Secondly, we aimed to assess whether these relationships are influenced by sex and exhibit regional specificity.

## Results

### Sex differences in muscle architecture

**Figure 1:**
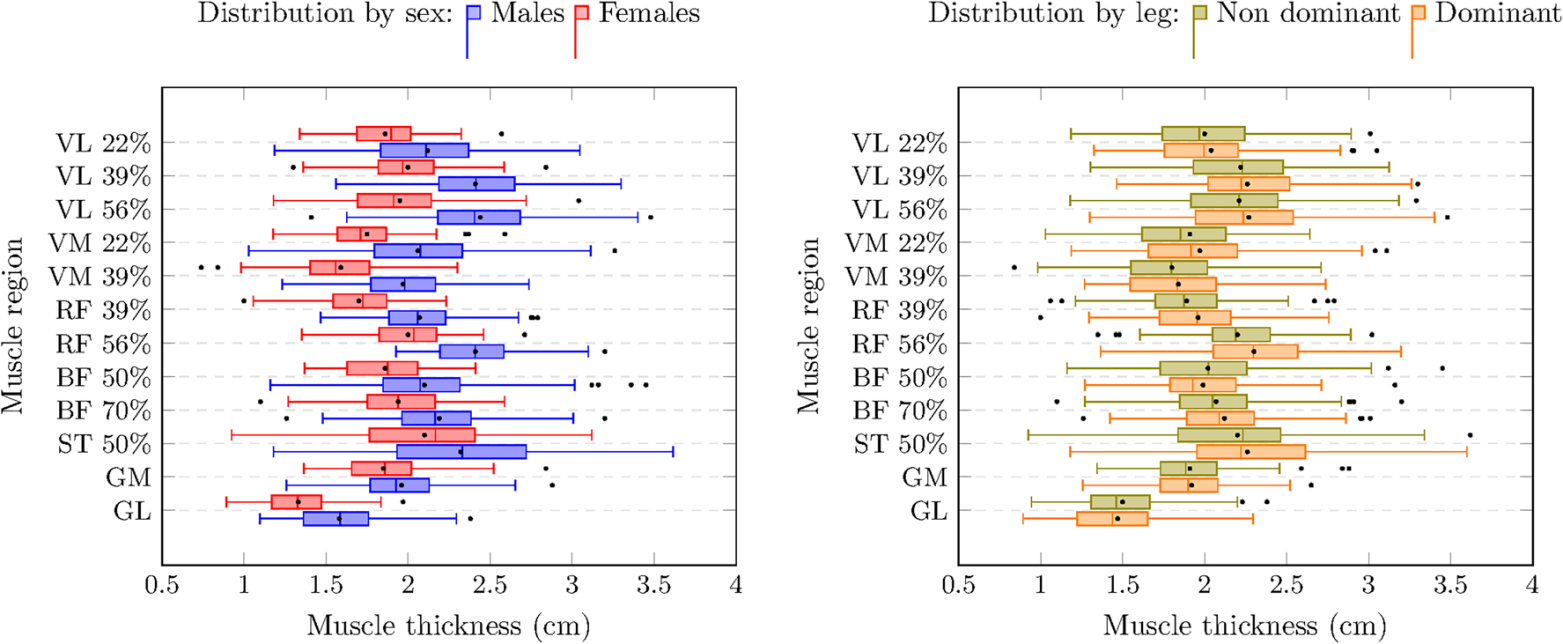
Muscle thickness distribution by sex and leg dominance. The left and right sides of the box correspond to the first (Q1) and third (Q3) quartiles, respectively. The central line indicates the median. The left whisker delimits the smallest data point greater than or equal to Q1 − 1.5 * (Q3–Q1). The right whisker delimits the largest data point less than or equal to Q3 + 1.5 * (Q3–Q1). Inside the boxplot, between Q1 and Q3, the mean value is shown with a black dot. See Table 1 for statistical analyses.

**Figure 2:**
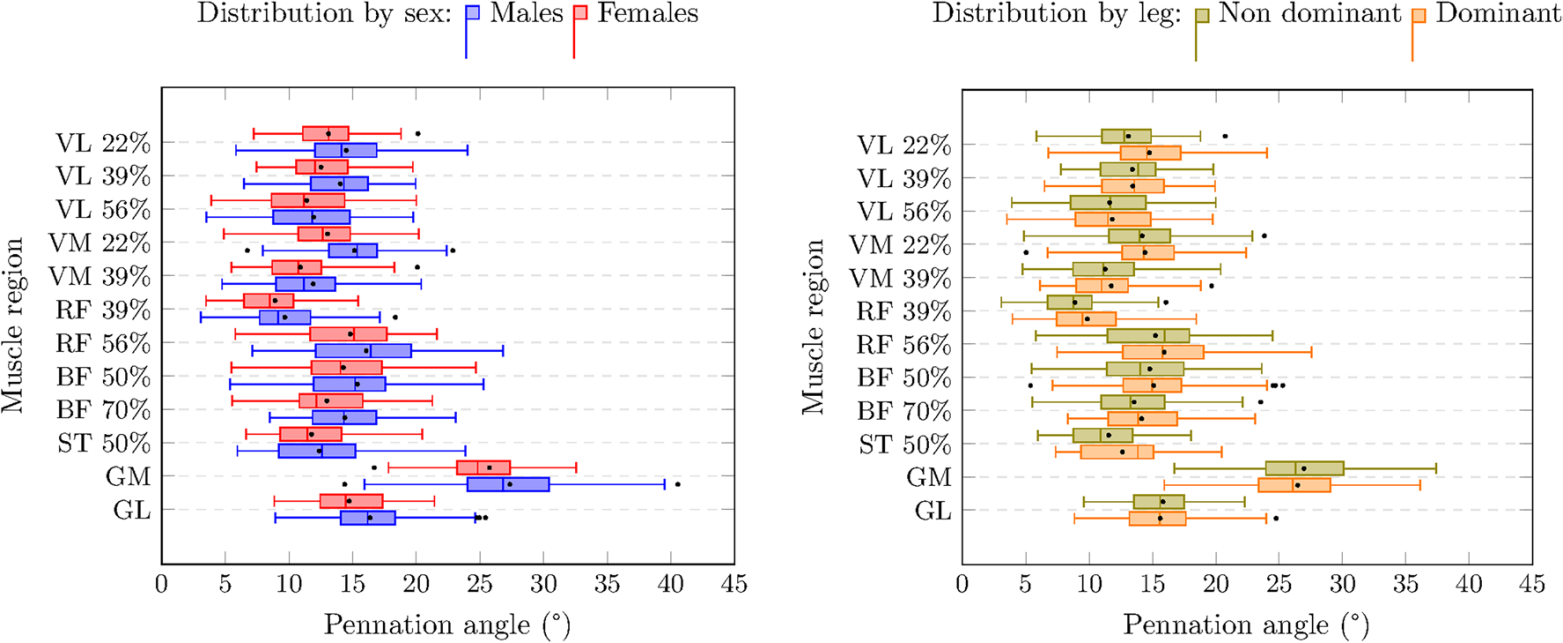
Pennation angle distribution by sex and leg dominance. The left and right sides of the box correspond to the first (Q1) and third (Q3) quartiles, respectively. The central line indicates the median. The left whisker delimits the smallest data point greater than or equal to Q1 − 1.5 * (Q3–Q1). The right whisker delimits the largest data point less than or equal to Q3 + 1.5 * (Q3–Q1). Inside the boxplot, between Q1 and Q3, the mean value is shown with a black dot. See Table 1 for statistical analyses.

**Figure 3:**
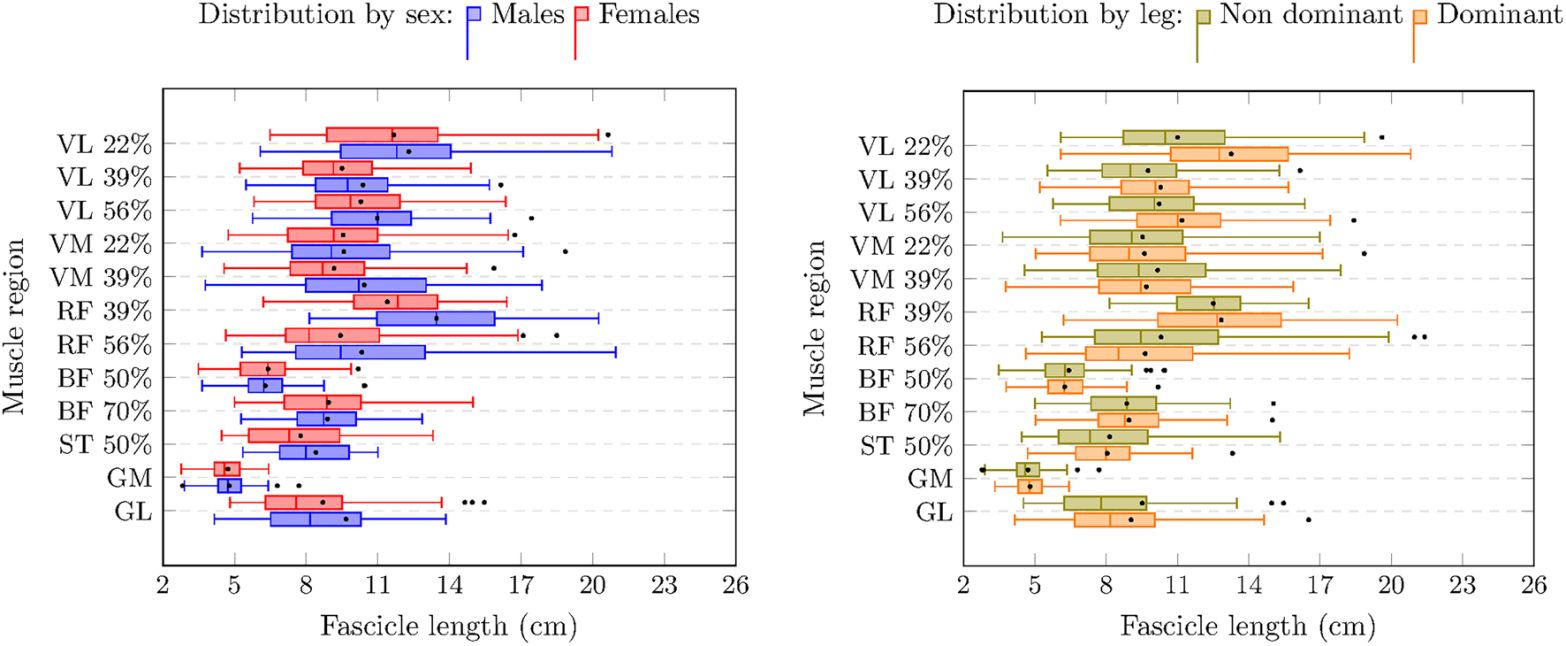
Fascicle length distribution by sex and leg dominance. The left and right sides of the box correspond to the first (Q1) and third (Q3) quartiles, respectively. The central line indicates the median. The left whisker delimits the smallest data point greater than or equal to Q1 − 1.5 * (Q3–Q1). The right whisker delimits the largest data point less than or equal to Q3 + 1.5 * (Q3–Q1). Inside the boxplot, between Q1 and Q3, the mean value is shown with a black dot. See Table 1 for statistical analyses.

In all muscles, males had greater MT compared to females (from 0.1 cm in GM to 2.1 cm in GL, *p* < 0.01) (Table 1). In the VL, sex differences in MT were moderated by the region measured within the muscle (from distal to proximal the differences were 0.3 cm, 0.4 cm, and 0.5 cm, all *p* < 0.001). For all muscles, there were no sex differences in FL. In terms of PA, males had wider angles than females in the VL (1.3°, p = 0.02), GM (2.1°, *p* = 0.01), and GL (1.2°, *p* < 0.001). The dominant leg had higher MT (0.1 cm, *p* = 0.01) and PA (1.5°, *p* = 0.01) than the non-dominant one in the RF. In the VL, both FL and PA were higher in the dominant than the non-dominant leg (1.2 cm, *p* < 0.001; 0.6°, *p* = 0.02, respectively). The distribution by sex and legs regarding muscle architecture is displayed in Figs. 1, 2, 3.

**Table 1:** Sex, regional, and leg differences in muscle architecture.

Muscle	Mean ± SD			Sex differences			Regional differences			Leg differences		
	MT (cm)	FL (cm)	PA (°)	MT (cm)	FL (cm)	PA (°)	MT (cm)	FL (cm)	PA (°)	MT (cm)	FL (cm)	PA (°)
VL	2.2 ± 0.4	10.9 ± 3.1	13.1 ± 3.6	S x R	0.02	1.3^‡^	S x R	R^1^	R^2^	0.1	1.2^†^	0.6^‡^
VM	1.9 ± 0.4	9.7 ± 3	12.9 ± 3.8	0.3^†^	0.6	1.2	0.1	1.1	− 3.2	0.1	− 0.3	0.8
RF	2.1 ± 0.4	10.5 ± 3.6	12.5 ± 5	0.4^†^	0.9	0.2	0.3^†^	− 2.7^§^	6.7^†^	0.1^§^	0.5	1.5^§^
BFlh	2.1 ± 0.4	7.6 ± 2.1	14.4 ± 4.1	0.3^†^	0.1	0.7	0.1	2.6^†^	− 1.2	− 0.1	− 0.3	0.6
ST	2.2 ± 0.5	8.1 ± 2.4	12.1 ± 3.8	0.3^‡^	1.3	1.3	–	–	–	0.02	− 0.8	0.6
GM	1.9 ± 0.3	4.8 ± 0.8	26.7 ± 4.9	0.1^§^	0.1	2.1^§^	–	–	–	− 0.01	0.1	− 0.9
GL	1.5 ± 0.3	8.1 ± 1.9	15.7 ± 3.2	2.1^†^	0.7	1.2^†^	–	–	–	0.02	− 0.2	− 0.4

*MT* muscle thickness; *FL* fascicle length; *PA* pennation angle; *VL* vastus lateralis; *VM* vastus medialis; *RF* rectus femoris; *BFlh* biceps femoris long head; *ST* semitendinosus; *GM* gastrocnemius medialis; *GL* gastrocnemius lateralis. Sex differences were expressed as men–women. Regional differences were expressed as the proximal–distal regions in muscles with different regions (VM, RF, and BF). Leg differences were expressed as dominant leg–non-dominant leg. R^1^ = equal FL across regions; R^2^ = PA differences by region: 0.01 for the middle region–most distal region, − 2.3^†^ for the most proximal region–most distal region, and − 1.8^†^ for the most proximal region–middle region. S x R = interaction between sex and region in MT. A simple effect analysis of this interaction is explained in the results section. († = *p* < 0.001; ‡ *p* = 0.02; § *p* = 0.01).

### Muscle regional differences

The FL was longer in the proximal than the distal region (2.6 cm, *p* < 0.001) of the BFlh. In the RF, the proximal region had greater muscle MT (0.3 cm, *p* < 0.001), wider PA (6.7°, *p* < 0.001), and shorter FL (− 2.7 cm, *p* = 0.01) than the distal region. The VL showed regional homogeneity in FL, while in terms of PA, the distal and medial regions were homogeneous but with lower PA (− 1.8°, *p* < 0.001) in the proximal region. In terms of MT, the VL presented regional heterogeneity exhibiting morphological differences between males and females. Muscle thickness in males was 0.28 cm (*p* = 0.02) greater in the middle region than the most distal region, 0.33 cm (*p* < 0.001) greater in the most proximal than the most distal region, and 0.03 cm greater in the most proximal than the middle region. In females, the corresponding values were 0.13 cm (*p* < 0.001), 0.08 cm, and 0.05 cm, respectively. The VL, RF, and BFlh exhibited regional differences regarding MT, FL, and PA while the VM was homogeneous across its regions for all architectural variables (Table 1).

### Causal mediation analyses: mediation, moderated mediation, and mediated moderation

Overall, across subjects, an association between the increase in MT and the increase in PA, which eventually decreased due to an associated increase in FL. All direct and indirect effects were significant (all *p* < 0.05). Regarding total effects, all were significant (all *p* < 0.01), except for total effects in the distal and proximal regions of RF. The direct, indirect, and total effects are reported in sexagesimal angle per 1 mm increment in MT across subjects. In muscles that were measured regionally, the direct, indirect, and total effects were conditioned effects (based on the measured region).

In all muscles, a 1 mm increase in MT across subjects was associated to a significant increase (all *p* < 0.001) in PA (direct effect), ranging from 0.12° in the ST to 1.32° in the GM (Table 2). Interestingly, the increase in FL was also associated with an increase in MT across subjects. This circumstance caused a significant decrease (indirect effect) in PA (all *p* < 0.05) in all cases, ranging from 0.02° in the VL(22%) to 0.47° in the distal region (70%) of the BFlh. Therefore, the total effect in all cases was smaller than the direct effect due to the suppressive effect exerted by the increase in FL, ranging from 0.04° in RF(56%) to 0.88° in GM. At the group-level, the percentage of suppressive effect on PA due to the increase in FL when increasing MT by 1 mm. ranges from 5.2% in VL(22%) to 92.3% in RF(56%). Additionally, in the RF, the total effect was not significant, suggesting that changes in MT translated into changes in FL while maintaining PA invariant across subjects. These results remain consistent after adjusting for height in all muscles except for the total effect in the BFlh, which was not significant after accounting for height (Table 3).

**Table 2:** Causal mediation analyses adjusted by leg and sex.

Muscle	Region	Indirect effect (95% IC)	Direct effect (95% IC)	Total effect (95% IC)
VL	22%	− 0.027 [− 0.059, 0]^§^	0.514 [0.395, 0.63]^†^	0.487[0.364,0.61]^†^
	39%	− 0.058 [− 0.114, − 0.01]^§^	0.539 [0.424, 0.65]^†^	0.480 [0.358, 0.60]^†^
	56%	− 0.112 [− 0.182, − 0.05]^†^	0.512 [0.399, 0.62]^†^	0.401 [0.286, 0.52]^†^
VM	22%	− 0.127 [− 0.194, − 0.07]^†^	0.348 [0.245, 0.45]^†^	0.221 [0.101, − 0.23]^†^
	39%	− 0.246 [− 0.358, − 0.15]^†^	0.472 [0.331, 0.62]^†^	0.227 [0.068, 0.38]^‡^
RF	39%	− 0.292 [− 0.530, − 0.09]^‡^	0.464 [0.224, 0.71]^†^	0.173[− 0.117,0.46]
	56%	− 0.420 [− 0.6, − 0.24]^†^	0.455 [0.287, 0.62]^†^	0.035 [− 0.194, 0.26]
BFlh	50%	− 0.466 [− 0.679, − 0.27]^†^	0.714 [0.571, 0.87]^†^	0.248 [0.016, 0.49]^§^
	70%	− 0.13 [− 0.206, − 0.06]^†^	0.518 [0.382, 0.65]^†^	0.389 [0.240, 0.53]^†^
ST	50%	− 0.04 [− 0.07, − 0.01]^‡^	0.12 [0.09, 0.16]^†^	0.08 [0.04, 0.13]^‡^
GM	–	− 0.44 [− 0.63, − 0.25]^†^	1.316 [1.17, 1.45]^†^	0.88 [0.65, 1.09]^†^
GL	–	− 0.17 [− 0.27, − 0.1]^†^	0.43 [0.27, 0.6]^†^	0.27 [0.1, 0.5]^‡^

In those muscles where the indirect effects are shown by regions, the direct and total effects are conditional effects estimated for the specific region. *VL* vastus lateralis, *VM* vastus medialis, *RF* rectus femoris, *BFlh* biceps femoris long head, *ST* semitendinosus; *GM* gastrocnemius medialis, *GL* gastrocnemius lateralis. † = *p* < 0.001, ‡ *p* = 0.02, § *p* = 0.01.

**Table 3:** Causal mediation analyses adjusted for height, leg, and sex.

Muscle	Region	Indirect effect (95% IC)	Direct effect (95% IC)	Total effect (95% IC)
VL	22%	− 0.030 [− 0.062, − 0.01]^‡^	0.512 [0.401, 0.62]^†^	0.484 [0.377, 0.60]^†^
	39%	− 0.063 [− 0.117, − 0.02]^‡^	0.54 [0.44, 0.65]^†^	0.477 [0.364, 0.59]^†^
	56%	− 0.115 [− 0.184, − 0.05]^†^	0.513 [0.405, 0.62]^†^	0.398 [0.281, 0.52]^†^
VM	22%	− 0.131[− 0.206, − 0.07]^†^	0.345 [0.238, 0.45]^†^	0.214 [0.097, 0.34]^†^
	39%	− 0.248 [− 0.358, − 0.15]^†^	0.471 [0.331, 0.62]^†^	0.223 [0.069, 0.38]^‡^
RF	39%	− 0.296 [− 0.539, − 0.10]^†^	0.468 [0.243, 0.71]^†^	0.182 [− 0.095, 0.45]
	56%	− 0.427 [− 0.61, − 0.25]^†^	0.464 [0.298, 0.63]^†^	0.036 [− 0.208, 0.27]
BFlh	50%	− 0.464 [− 0.666, − 0.26]^†^	0.693 [0.556, 0.83]^†^	0.228 [− 0.0027, 0.47]
	70%	− 0.133 [− 0.214, − 0.06]^†^	0.498 [0.359, 0.63]^†^	0.367 [0.223, 0.51]^†^
ST	50%	− 0.042 [− 0.078, − 0.01]^‡^	0.123 [0.086, 0.13]^†^	0.08 [0.034, 0.13]^‡^
GM	–	− 0.431 [− 0.62, − 0.25]^†^	1.328 [1.19, 1.46]^†^	0.90 [0.69, 1.12]^†^
GL	–	− 0.158 [− 0.248, − 0.08]^†^	0.398 [0.232, 0.56]^†^	0.24 [0.066, 0.4]^‡^

In those muscles where the indirect effects are shown by regions, the direct and total effects are conditional effects estimated for the specific region. *VL* vastus lateralis, *VM* vastus medialis, *RF* rectus femoris, *BFlh* biceps femoris long head, *ST* semitendinosus; *GM* gastrocnemius medialis, *GL* gastrocnemius lateralis. † = *p* < 0.001, ‡ *p* = 0.02, § *p* = 0.01.

Among the muscles measured across multiple regions, the conditional indirect effects (the indirect effects of each muscle region) differ significantly only in the BFlh, as shown by the non-overlapping confidence intervals (Tables 2 and 3). This indicates that only in the BFlh was the indirect effect moderated by the muscle region (mediated moderation). Specifically, for this muscle, the 95% Bootstrap confidence interval for the difference in conditional indirect effects was [− 0.54, − 0.05], further evidencing the moderation. In all the mediator models tested, the interaction between muscle thickness and muscle region was not significant, indicating the lack of mediated moderation. Concerning the moderation of the direct effect by the muscle region, Tables 2 and 3 show all the confidence intervals for the conditional direct effects within each multi-regionally measured “muscle” almost entirely overlap. The latter indicates that the muscle region does not moderate the direct effects. Comparing GM with GL among subjects, a 1 mm change in MT had a significantly greater impact on the PA, which increased approximately threefold in the GM (1.316°/0.43°) than the GL. It is noteworthy that the GM exhibited the highest PA among the muscles and displayed the largest direct effect (Table 2). The results obtained in the causal mediation analysis were essentially similar when sex was excluded from the models.

## Discussion

**Figure 4:**
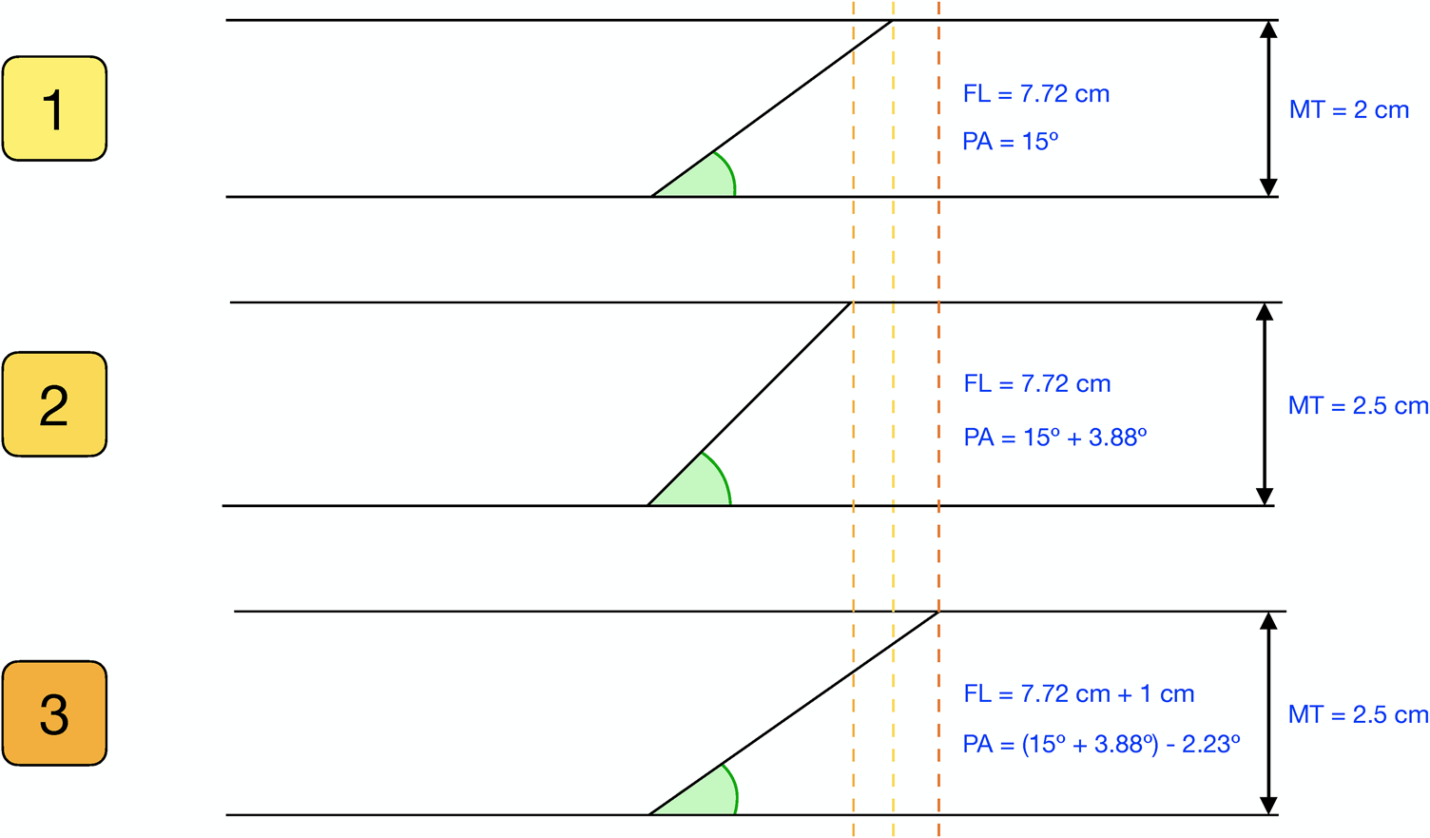
Different scenarios of the mediation model. 1 = first scenario, muscle initial state; 2 = second scenario, where the pennation angle increases if fascicle length does not change after an increase in muscle thickness (the direct effect accounts for this fact); 3 = third scenario, where the pennation angle decreases due to fascicle length increment (the direct effect partly cancels the direct effect). *MT* muscle thickness; *PA* pennation angle; *FL* fascicle length.

This investigation revealed through modelling that an increase in MT is associated with a widening of the PA, but this effect is influenced by changes in FL, suggesting a suppressive effect of FL on PA in all muscles. Furthermore, this study sheds light on architectural differences across several dimensions: sex, leg dominance, specific muscles, and within individual muscles, specific regions—thereby extending prior knowledge^13–16^. Overall, these findings indicate that the interplay between MT, PA, and FL is specific for different muscles and within a given muscle shows a regional variation (Fig. 4), as discussed elsewhere^5,7,8^.

In all muscles, males had greater MT compared to females (from 0.1 cm in GM to 2.1 cm in GL), as previously reported^17^. The difference in MT between males and females in lower limb muscles could be primarily influenced by biological and physiological factors, including sex hormones, muscle fibre type distribution, and overall body composition. In terms of sex hormones, males typically have significantly higher levels of testosterone compared to females^18^, which can lead to greater muscle hypertrophy and thus increased MT^19^. While MT and FL are related, they are not entirely dependent on each other. In agreement with studies performed using diffusion-tensor magnetic resonance imaging, we found no sex differences regarding FL in all muscles^20^. FL is influenced by factors such as tendon length, joint structure, limb proportions, and age^20–22^. Our study also highlighted sex differences in VL, GM, and GL regarding PA (i.e., males > females), which is also in line with the literature^17,23,24^. Males are taller than females, which has been genetically explained elsewhere^25,26^, so could that alone explain the differences in muscle architecture? While body height could indeed influence certain aspects of muscle architecture, it is not the sole determinant of the observed differences between males and females. Body height could affect absolute muscle size, as larger bodies generally need larger muscles to support and move them. However, our results indicate that when it comes to relative muscle architectural features, body height alone is insufficient to explain the observed differences. This is supported by the fact that after accounting for height in our analyses the results remain essentially unchanged. In agreement, numerous studies have shown that even when adjusted for body size, males typically have greater muscle mass than females, suggesting that factors beyond body size, such as hormonal differences, are contributing to these disparities^27,28^. For example, sex differences in muscle fibre types^29^ could also explain sex differences in muscle architecture, although this possibility remains unexplored. Since the observed sex differences remained primarily unchanged after accounting for height, the present findings indicate that height plays a minor role in the observed muscle architectural differences between males and females in the present investigation. Moreover, the results obtained in causal mediation analysis were essentially similar after excluding sex, indicating that the relationships described are robust and similar in males and females.

In terms of leg differences due to dominance, our data show higher MT and PA in the dominant than non-dominant leg in the RF. Additionally, the VL showed higher FL and PA in the dominant leg. Such differences could be attributed to the increased mechanical loading^30^ and functional demands^31^ of the dominant leg vs. the non-dominant leg. In terms of regional differences, the VL, RF, and BFlh exhibited differences regarding MT, FL, and PA while the VM was homogeneous across its regions for all architectural variables. Our VL and RF results concur with Blazevich, et al.^13^. However, in contrast with our findings, these authors found regional differences in MT and PA of the VM. This discrepancy could be attributed to several factors, such as differences in the study population, equipment used, and imaging acquisition techniques.

In the resting BFlh, non-significant differences in FL between the proximal and distal regions have been reported (~ 7.4 ± 0.5 cm, and ~ 6.4 ± 1 cm, respectively)^32^ using real-time ultrasound. The latter agrees with data collected from cadavers although limited to elderly males and females (> 80 years)^16^. However, certain cadaveric studies involving individuals aged over 65 years have revealed longer FL proximally than distally (~ 7.1 ± 0.5 cm, and ~ 6.4 ± 0.9 cm, respectively). These regional differences could be attributed to anatomical constraints (e.g., the insertion points of tendons or the shape of the bone attachments)^33^, functional requirements of daily life or during exercise^34^, and mechanical loading^35^. Moreover, it has been suggested that the central nervous system may independently control different regions of the BFlh^36–39^. In agreement, it has been reported that this muscle is innervated by more than one motor nerve branch^40^, allowing a task-specific activation of different regions^41^. The present study is one of the few investigations that has measured the architecture of the BFlh throughout its length in a large sample of volunteers^14,32,42^. Detailed examination of the architectural arrangement of the fibres along muscle length will allow a better understanding of BFlh functional properties.

In our analysis using pooled data, our model predicts that increasing the MT by 1 mm while keeping the FL unchanged should result in a significant widening of the PA in all muscles This increase ranged from 0.12° in the ST to 1.32° in the GM. Nevertheless, the FL increases with muscle thickness. Consequently, our model predicts that a FL increment should be associated with a concurrent decrease in the PA. The term to describe this phenomenon within the field of mediation analyses is defined as “suppression”^43^. Suppression refers to a phenomenon wherein a single causal variable exhibits a relationship with an outcome variable through two distinct mediator variables, with one mediated effect being positive and the other negative. In such instances, each mediator variable suppresses or masks the effect that is transferred through the other mediator variable^43^. These results are in line with a previous investigation of our group which revealed this phenomenon in the tibialis anterior muscle^12^. However, in the RF, our modelling results indicate that concurrent increases in MT and FL should result in no significant alterations in PA. This finding can be attributed to the RF’s anatomical arrangement as a fusiform muscle, characterized by its parallel arrangement of muscle fibres.

Some studies have reported that resistance-trained individuals, such as bodybuilders or rugby players, exhibit larger MT and PA compared to untrained individuals, but no significant differences in FL. These findings suggest that FL may not increase with resistance training^8^. The question of whether adaptations to such stimuli manifest in an increase in FL remains a subject of controversy and ongoing debate among scholars. However, it has been observed that the FL may indeed increase with exercise training, depending on the type of muscle contraction (i.e., eccentric, or concentric) involved^4,7^. Additionally, studies have shown that FL is larger in powerlifters^44^ and sumo lifters^45^. A study on untrained males^46^ showed that, although there was a significant increase in the average cross-sectional area of muscle fibres and PA after resistance training, changes in FL were not significant. These findings again suggest that adaptations in FL may not be a primary contributing factor to training-induced muscle hypertrophy. The observation that specific types of exercise training can lead to increases in both MT and FL aligns with our mediation model. Furthermore, recent insights from a study by Hornberger et al.^47^ offer a mechanistic explanation for the observed sex differences in MT and PA. According to their findings, mechanical loading induces changes in fascicle length and diameter, leading to alterations in whole-muscle CSA. In males, resistance training may elicit greater adaptations in fascicle length and diameter compared to females, resulting in larger MT and PA. Specifically, longitudinal growth of fascicles contributes to increased MT, while radial growth leads to a larger PA. Therefore, the observed sex differences in MT and PA could be attributed to the differential response of muscle fascicles to mechanical stimuli between males and females, wherein males may exhibit more pronounced adaptations favouring muscle hypertrophy, which is better captured by MT.

The relationship between MT, FL, and PA is not always direct or causal, and further explanation is required. As previously mentioned, an increase in MT through resistance training does not necessarily entail a direct increase in other architectural features as reviewed by Kruse, et al.^5^. Increasing PA allows for an expansion of the physiological cross-sectional area, and consequently, enhances maximal force-generating capacity^48,49^. However, with an increased PA, the force transmitted along the line of action of the muscle by each fibre decreases^50,51^. Nonetheless, despite the less efficient force transfer per muscle fibre, a greater PA enables more muscle fibres to attach to the tendon compared to a parallel muscle^52^ or an increase in the amount of myofiber within each fibre, thereby allowing for the generation of greater force. On the other hand, fibre-type composition could affect muscle architecture features. Slow-twitch (Type I) and fast-twitch (Type II) fibres exhibit distinct contractile properties and metabolic profiles. Muscles with a higher proportion of fast-twitch fibres may exhibit greater MT due to their potential for greater hypertrophy in response to resistance training^53,54^. Additionally, variations in FL and PA may also be influenced by muscle fibre composition. Fast-twitch fibres are typically associated with shorter FL and a greater PA, which can contribute to increased force production^22,55^. However, the mechanisms underlying the relationship between muscle fibre composition and architectural characteristics warrant further investigation to elucidate their interplay fully.

Lastly, the BFlh exhibited moderated mediation, showing FL differences between its regions. In this regard, a noteworthy 21% increase in BFlh FL after three weeks of eccentric training has been observed in the distal compared to the central region^34^. This finding aligns with emerging evidence suggesting that muscle growth is not uniform throughout the entire muscle, as supported by recent studies^4,56–58^. The mechanism has been attributed to a heterogeneous distribution of fibre strain^59^ and muscle activity^60^ along the BFlh. The present findings are based on the overall inter-individual heterogeneity and individual departure from the mean is possible^61^. However, our mediation analysis is robust, suggesting that this could be true for pennate muscles but not for parallel muscles such as the RF.

The main strengths of this study are the large number of subjects (n = 109), the inclusion of males and females of similar age, and the employment of robust statistical methods. In addition, we employed 2-D ultrasound (B-mode) to delineate muscle architecture, the most common technique used for this purpose in both cross-sectional and longitudinal studies. This study has also limitations, which mainly relate to its cross-sectional design, limiting the extrapolation of our results from the group to the individual. Although we used modern 2D-ultrasound equipment, it is worth mentioning that current state-of-the-art 3D techniques such as diffusion tensor imaging allow an objective measurement of the PA and FL, avoiding some of the limitations associated with current 2D technology^2^. For example, part of the length of the FL had to be estimated, which entails an additional error of measurement for this specific variable. However, the impact of this estimation-associated error on FL assessment should have been similar across subjects, as suggested by the fact that our main conclusion agrees with that reported using diffusion-tensor magnetic resonance imaging^20^. An estimation of the average error using the CMA approach in 2D compared to more direct measurement (e.g., extended field of view or 3D techniques) should be analysed in future studies to consider the curvature of the FL. Future randomized controlled designs should be carried out with designs including concentric and eccentric training groups, as well as controls to verify our results accounting for the complex interplay between MT, PA, and FL. Moreover, studies analysing the effects of muscle atrophy would also add validity to the present findings. Lastly, normalized FL (fascicle length/limb length) should be included in future analyses to control differences in FL produced by differences in limb size.

This study unveils significant intramuscular and intermuscular variations in human muscle architecture, highlighting the intricate dynamics among muscle thickness, pennation angle, and fascicle length. Notably, substantial sex-related differences were observed, which cannot be attributed to sex differences in height. Males consistently exhibited greater muscle thickness across all muscles. Regarding the pennation angle, males revealed wider angles than females in the vastus lateralis, as well as the gastrocnemius medialis and lateralis. However, there were no discernible differences in fascicle length between the sexes. Our study also revealed a suppressive effect of fascicle length on the pennation angle of lower limb pennate muscles. Notably, this suppressive effect was found to be regionally moderated in the biceps femoris long head, wherein distinct differences in fascicle length were observed among its regions. Conversely, in the rectus femoris, concurrent increases in muscle thickness and fascicle length were observed without alterations in pennation angle. This finding can be attributed to the rectus femoris’ anatomical arrangement as a fusiform muscle, characterized by its parallel distribution of muscle fibres.

## Methods

### Study design and subjects

This cross-sectional study comprises two separate measurement sessions. The first measurement session was conducted to perform pre-tests, as previously reported^12^. In a second visit, the subject’s knee extensors, knee flexors, and plantar flexors were explored by ultrasound. A total of 109 physically active and healthy males (n = 64, 59%) and females (n = 45, 41%) volunteered to participate in the study. The descriptive characteristics of the study population, e.g., the body heigh of the subjects, and the inclusion criteria for participation in the study have been reported elsewhere^12^. The volunteers were physically active, engaging in 3 to 8 h of moderate-intensity physical activity weekly. Several participants had a varied athletic background, having participated in various sports throughout their careers. However, the majority had, at some point, played soccer.

Self-selected limb dominance was determined by asking the participants which is their preferred leg to kick a ball as far as possible^62^. Most male (80%) and female (96%) subjects reported right-leg dominance. A written informed consent was obtained and signed by all volunteers after receiving information about the aims and potential risks of the study. The study commenced after approval by the Ethical Committee of the University of Las Palmas de Gran Canaria (CEIH2017/13) and was carried out according to the Declaration of Helsinki. The sex and gender of the subjects were defined based on self-reports during subject recruitment, and all subjects were reported as cisgender.

### Ultrasound imaging

**Figure 5:**
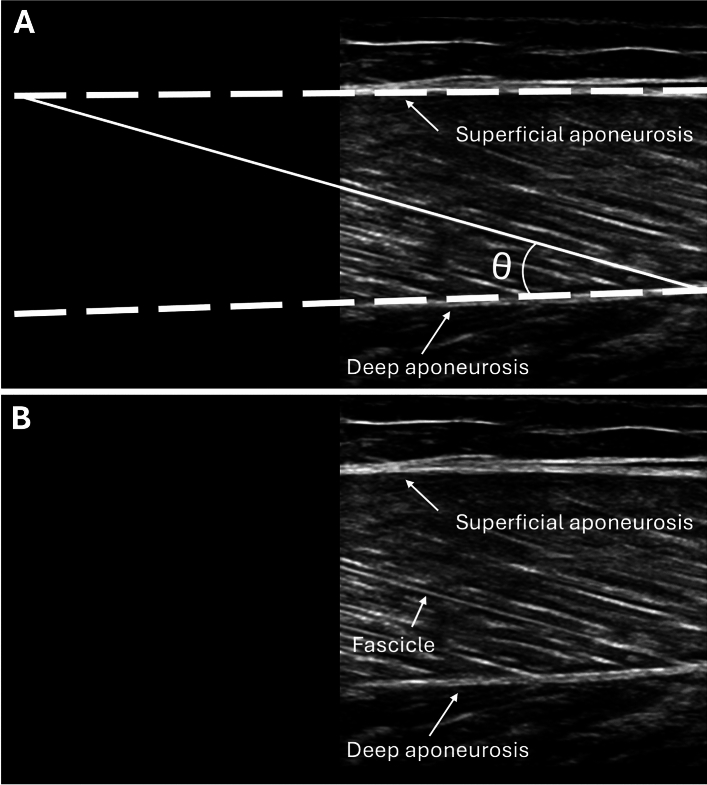
A demonstrative ultrasound image corresponding to the gastrocnemius lateralis of a male participant is shown. The white straight line indicates a fascicle, and θ shows the pennation angle. (**A**) Fascicle length was calculated by linear extrapolating the visible portion of fascicles to the intersection point with the linearly projected superficial muscle aponeurosis. (**B**) Original ultrasound image.

Real-time two-dimensional B-mode ultrasound (Philips CX50, Philips Medical Systems, Netherlands) with a 38 mm linear-array transducer (12–3 MHz, L12-3 Broadband, Phillips), was used to bilaterally measure the muscle architecture of the knee extensors (rectus femoris, vastus medialis, and lateralis; RF, VM, and VL, respectively), knee flexors (biceps femoris long head and semitendinosus; BFlh and ST, respectively), and plantar flexors (gastrocnemius medialis and lateralis; GM and GL, respectively). All scans started after 15 min of the subject´s lying supine on a gurney fully relaxed to allow completion of fluid shifts during changing from the upright position. Image acquisition was performed by an operator with extensive experience in musculoskeletal ultrasonography. Current guidelines and recommendations for musculoskeletal ultrasonography by the European Federation of Societies for Ultrasound in Medicine and Biology were followed^63^. Depending on the subject, the ultrasound depth and frequency were adjusted to 4–5 cm and 38–41 Hz (knee extensors and plantar flexors), while 5–6 cm and 36–38 Hz (knee flexors). The probe was hand-held, and the measurements were made with the subject in a prone position or supine position, depending on the analysed muscle, checking joint angles with a manual goniometer when was necessary. The ultrasound probe was placed perpendicular to the skin and parallel to the muscle fascicles. A water-soluble gel was applied on the skin to obtain a high-resolution image without losing the detailed anatomical features of the muscles^64^. Each measurement site was marked on the skin surface with a surgical pen to ensure that the probe was placed in the proper position. Using the gel meant that the ultrasound probe could be positioned just above the skin surface at each landmark without pressure being applied to the skin. The primary inclusion criterion for ultrasound image analyses was that the aponeuroses were as parallel as possible since the angle between the superficial and the intermediate aponeuroses can strongly influence the extrapolation methodologies^2,13^. A representative in-house image from the ultrasound data collected is presented in Fig. 5.

### Muscle architecture assessment

In each muscle, MT was measured as the distance between the superficial and deep aponeuroses at both the beginning and the end of the image, and the average of these distances was taken as the representative value. In addition, the PA and FL were each measured three times at different points along the ultrasound image of the muscle region, and the averages of these measurements were calculated to obtain representative values. Since the muscle´s fascicles were longer than the width of the probe, FL was calculated by linear extrapolation of the visible portion of fascicles to the intersection point with the linearly projected superficial aponeurosis of the muscle^65^. The inclusion criteria for determining appropriate fascicles to analyze were the following: the fascicle insertion point into the central aponeurosis must have been visible, and a reasonable portion of the fascicle (~ 25% or more of the total estimated length) must have been visible within the ultrasound transducer’s field of view^66^. Muscle architectural parameters (MT, PA, and FL) were digitized using image-processing software (OsiriX™ DICOM viewer, Pixmeo, Geneva, Switzerland). Overall, 2616 images and 20.928 measures (96 measures per leg) were recorded in all subjects. Ultrasound reliability was tested in four males before the start of the study. In brief, the operator acquired one image of all the muscles of each male at rest in the morning, in a relaxed state and without having exercised or done any vigorous activity in the previous 72 h. A person other than the operator segmented the images taken that day without knowing to whom each image belonged, that is, the images were blinded. This exact procedure was performed three days later. The intraclass correlation coefficient (ICC 3.1) and the confidence interval of each muscle are shown in Table 4. The latter is in line with the literature^67^, and it has been described according to a reference guideline for selecting and reporting for reliability research^68^.

**Table 4:** Intraclass correlation coefficients with 95% confidence intervals of ultrasound measurements.

Muscle	Region	Muscle thickness	Pennation angle	Fascicle length
VL	22%	0.998 [0.995, 0.999]	0.827 [0.573, 0.936]	0.977 [0.935, 0.992]
	39%	0.984 [0.954, 0.994]	0.875 [0.679, 0.954]	0.827 [0.574, 0.936]
	56%	0.929 [0.808, 0.974]	0.808 [0.533, 0.928]	0.924 [0.796, 0.973]
VM	22%	0.997 [0.991, 0.999]	0.975 [0.929, 0.991]	0.975 [0.929, 0.991]
	39%	0.99 [0.971, 0.996]	0.946 [0.853, 0.981]	0.938 [0.833, 0.978]
RF	39%	0.999 [0.998, 1]	0.944 [0.847, 0.98]	0.989 [0.732, 0.963]
	56%	1 [0.999, 1]	0.952 [0.869, 0.983]	0.951 [0.865, 0.983]
BFlh	50%	0.996 [0.998, 0.999]	0.849 [0.62, 0.944]	0.936 [0.828, 0.977]
	70%	0.996 [0.988, 0.999]	0.939 [0.835, 0.978]	0.836 [0.593, 0.939]
ST	50%	0.999 [0.997, 1]	0.972 [0.922, 0.99]	0.983 [0.952, 0.994]
GM	–	0.999 [0.996, 1]	0.935 [0.826, 977]	0.962 [0.895, 987]
GL	–	0.813 [0.543, 0.93]	0.929 [0.809, 0.975]	0.782 [0.482, 0.918]

*VL* vastus lateralis; *VM* vastus medialis; *RF* rectus femoris; *BFlh* biceps femoris long head; *ST* semitendinosus; *GM* gastrocnemius medialis; *GL* gastrocnemius lateralis.

### Knee extensors

**Figure 6:**
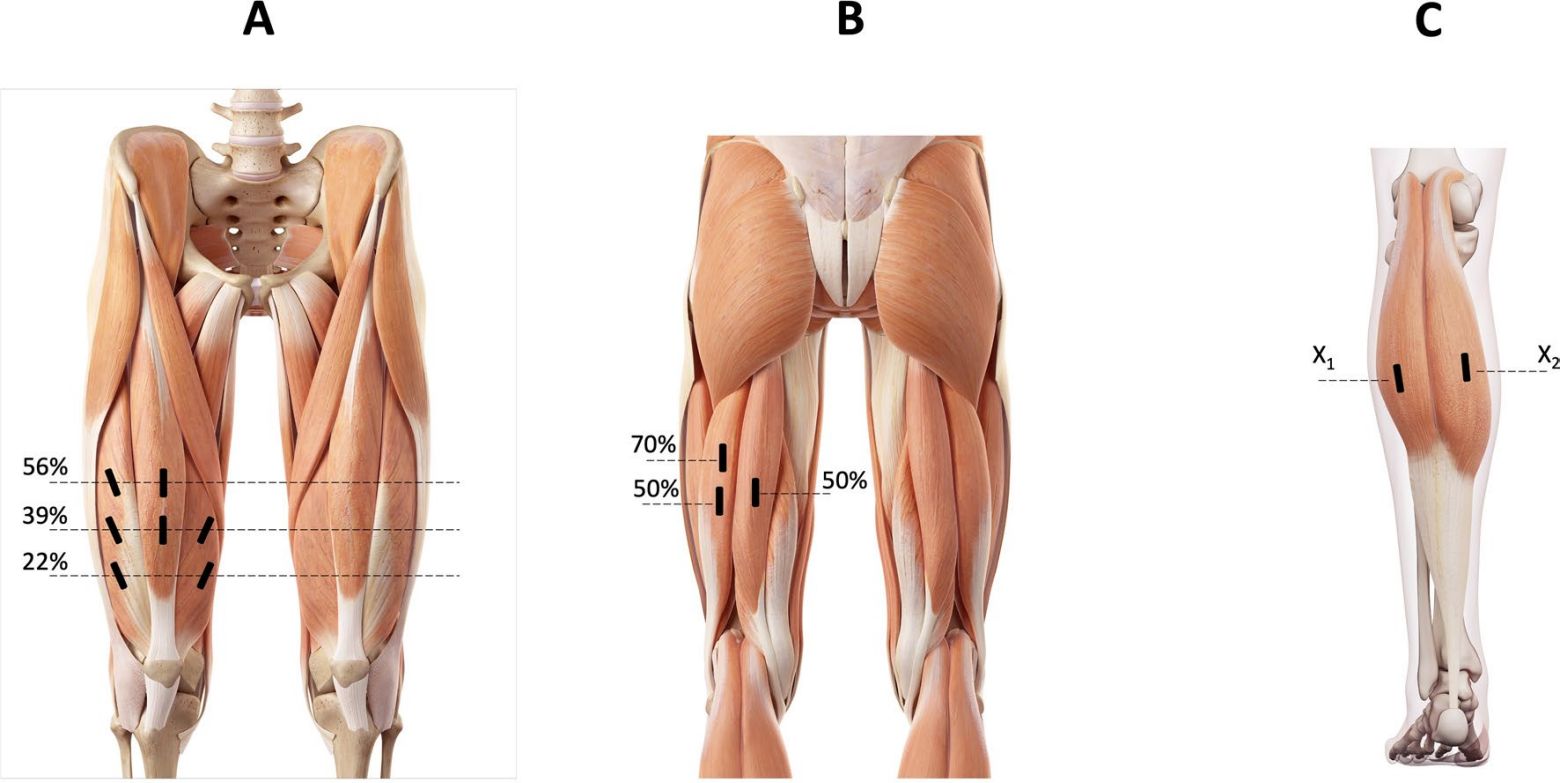
Muscle’s scanning sites. Muscle’s scanning sites. X1 = measurement zone for the gastrocnemius medialis; X2 = measurement zone for the gastrocnemius medialis. (**A**) vastus lateralis, medialis and rectus femoris: (**B**) biceps femoris long head and semitendinosus; (**C**)  gastrocnemius medialis and lateralis.

The subjects laid supine, their knees flexed to 45°, legs supported, and muscles relaxed. To standardize the ultrasound probe positions, the thigh length was measured from the superior border of the patella to the anterior superior iliac spine. Distal to proximal anatomical landmarks were marked upon the skin at 22, 39, and 56% of the measured length^13^. Ultrasound images of the RF (39 and 56%), VL (22, 39, and 56%), and VM (22 and 39%) were captured for later analysis (Fig. 6a).

### Knee flexors

The subjects laid prone with the hip and knee angles at 0° (full extension). To standardize the ultrasound probe positions, the common proximal BFlh and ST tendon at the ischial tuberosity and the distal myotendinous junctions were determined and marked on the skin, as reported^42^. Ultrasound images of BFlh and ST were taken at 50% and 70% along the line from the measured distal to proximal anatomical landmarks (Fig. 6b).

### Plantar flexors

The subjects laid prone with feet overhanging the gurney’s edge. To standardize the ultrasound probe position for the GM, the insertion on the medial condyle of the femur and the distal end of the muscle belly was determined and marked on the skin. Ultrasound images were obtained on the mid-longitudinal axis at two-thirds of the measured muscle belly length from the origin^69^. For the GL, images were acquired proximally, at 30% of the distance between the knee joint interline and the centre of the lateral malleolus, as previously reported^70^ (Fig. 6c).

### Statistical analysis

For each muscle, the mean and standard deviation (SD) of the overall sample is presented. A mixed-effects model was used to investigate differences among sexes, legs (dominant vs. non-dominant), and muscle regions (distal and proximal regions in BFlh, RF, VM, and three regions—distal, medial, and proximal—in the VL) in each parameter of muscle architecture. The subjects were considered random factors, while the complete model included fixed factors such as sex, leg, and region. For muscles measured in a single region (GM, GL, and ST), the same procedure was followed, excluding the region variable. In cases where a significant interaction was found, a simple effects analysis was performed using the “emmeans” package for R^71^.

**Figure 7:**
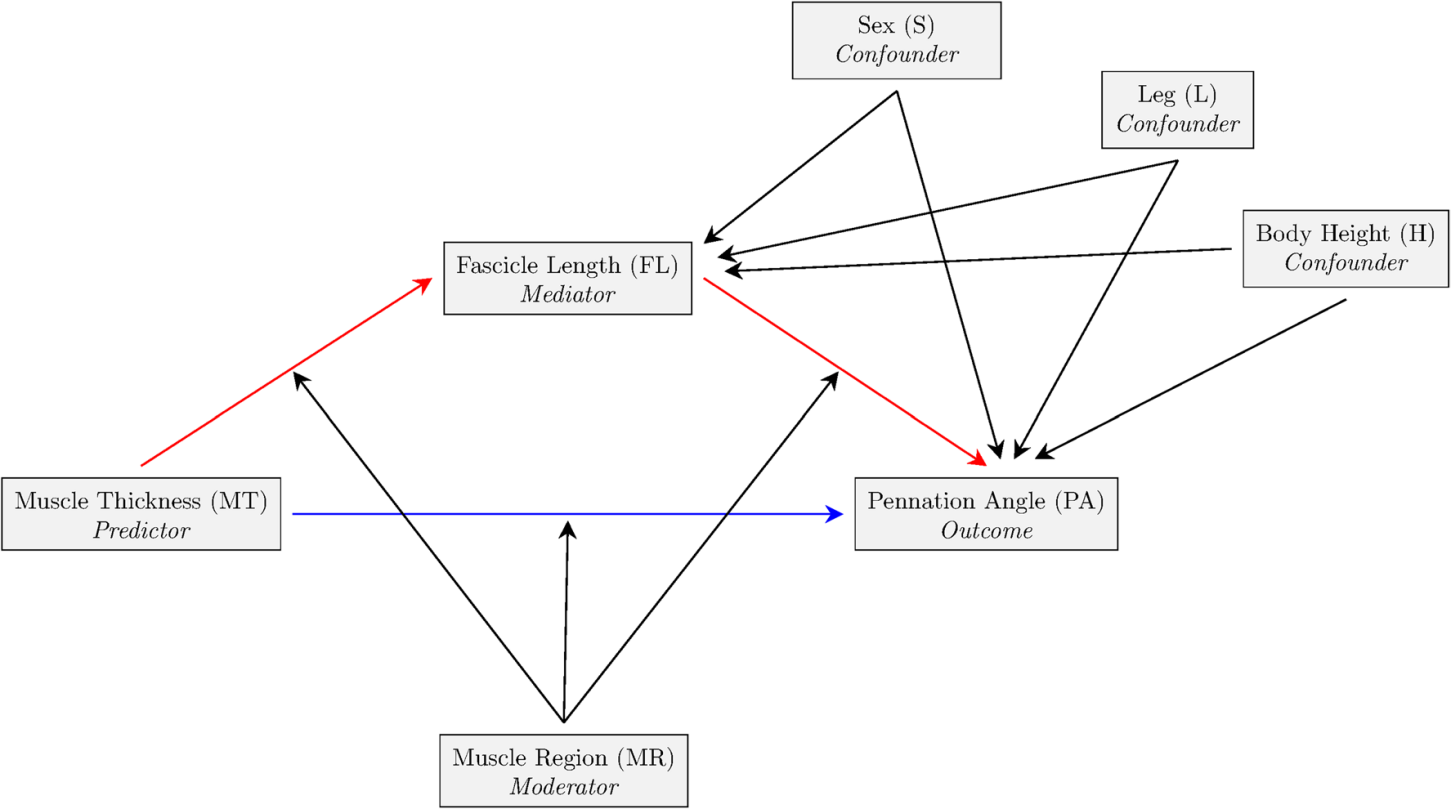
Mediation model diagram for muscle architecture variables taken from multiple regions in different muscles. For single region measurement, remove the node representing the muscle region variable and all its associated edges. The red edges indicate the pathway through which the indirect effect is exerted, whereas the blue edge indicates the pathway for the direct effect.

A mediation analysis for mixed models was conducted for each muscle (Fig. 7). For muscles with different regions measured within the same muscle (VL, VM, RF, and BFlh), the fixed part of the mediator model used FL as the dependent variable and was modelled as a linear mixed model. The model included adjustments for leg, region, sex, body heigh, and the interaction between region and MT (for the study of mediated moderation and moderated mediation). The outcome model, which used PA as the dependent variable, was also a linear mixed model and included MT and the mediator (FL), adjusted for leg, region, sex, body heigh, and the interactions: region x MT, as well as region x FL (for the study of mediated moderation and moderated mediation). Both the mediator and outcome models included random intercepts (i.e., subjects). For muscles without more than one region, the same procedure was followed, but the region variable was omitted in all models. In all cases, the estimated direct, indirect, and total effects were calculated for each 1 mm increment in MT. It is worth mentioning that in muscles measured at different regions along their length, the effects (direct, indirect, and total) are conditioned effects. The mediation analysis was performed using the “mediation” package for R^72^ (for further details, see Supplementary statistical methods). Due to the limitations of the “mediation” package in studying moderated mediation and mediated moderation, which can be induced by the region variable in muscles with different regions, bootstrap methods for mixed models were employed^73^. To assess whether the mediation analysis was influenced by sex, the model was run again after excluding sex.

Our mediation analyses adhere to the AGReMA statement (A Guideline for Reporting Mediation Analyses) for randomized controlled trials and observational studies^74^. The corresponding AGReMA checklist is provided as Supplementary information.

All statistical analyses were performed using R 4.2.2 (R Foundation for Statistical Computing, Vienna, Austria). When multiple comparisons were necessary, p-values were adjusted using the Bonferroni correction. Considering the sample size of this study (n = 109), we applied the central limit theorem and deemed the data suitable for parametric statistics. Statistical significance was defined as *p* < 0.05.

### Supplementary Information

Supplementary Information 1.

Supplementary Information 2.

Supplementary Information 3.

## Data Availability

The data that support the findings of this study are available from the corresponding author upon reasonable request.
