# Supplementary material for: The relationship between muscle thickness and pennation angle is mediated by fascicle length in the muscles of the lower extremities
Source: Sci Rep. 2024 Jun 27;14:14847. doi: 10.1038/s41598-024-65100-6 (PMC11211461; doi:10.1038/s41598-024-65100-6)
Supplement: Supplementary file 1 — Supplementary Information 1. [file 41598_2024_65100_MOESM1_ESM.docx]

**Supplementary files**

**Supplementary information.** A Guideline for Reporting Mediation Analyses (AGReMA).

**Supplementary statistical methods.** Description of the mathematical models and R code.
