# Supplementary material for: The relationship between muscle thickness and pennation angle is mediated by fascicle length in the muscles of the lower extremities
Source: Sci Rep. 2024 Jun 27;14:14847. doi: 10.1038/s41598-024-65100-6 (PMC11211461; doi:10.1038/s41598-024-65100-6)
Supplement: Supplementary file 3 — Supplementary Information 3. [file 41598_2024_65100_MOESM3_ESM.pdf]

Mixed Linear Models for Mediation Analysis

Below, we present the mediator and outcome models of the proposed mediation framework. These models can be derived from the relationships between the variables depicted in the mediation model diagram (Figure 7). The direct, indirect, and total effects are estimated by combining both models using the `mediate` function from the R statistical package `mediation`. For the muscles measured in multiple regions, these effects are conditional as the region acts as a moderating variable. At the end of the mathematical description of the mediation model, we include the R code for estimating the conditional effects of the vastus medialis in Table 3, which was measured in two regions (22% and 39%). A similar R code would apply to other muscles assessed in multiple regions to estimate the conditional direct, indirect, and total effects. For those muscles where the muscle architecture was measured in a single region (GM, GL, ST), it is sufficient to replace MT*MR with MR and FL*MR with MR in the R code and to remove the variable MR and all its interactions from the mathematical equations. Additionally, to estimate the direct, indirect, and total effects in Table 2, remove the confounding variable body height (H) from all equations.

Mediator Model:

$$\begin{aligned} FL_{ijk} = \alpha_0 + \alpha_1 MT_{ijk} + \alpha_2 MR_k + \alpha_3 (MT_{ijk} \times MR_k) \\ + \alpha_4 H_i + \alpha_5 S_i + \alpha_6 L_j + u_i + \epsilon_{ijk} \end{aligned} \tag{1}$$

Outcome Model:

$$\begin{aligned} PA_{ijk} = \beta_0 + \beta_1 MT_{ijk} + \beta_2 FL_{ijk} + \beta_3 MR_k \\ + \beta_4 (FL_{ijk} \times MR_k) + \beta_5 (MR_k \times MT_{ijk}) \\ + \beta_6 H_i + \beta_7 S_i + \beta_8 L_j + v_i + \eta_{ijk} \end{aligned} \tag{2}$$

where:

- FL_{ijk} is the fascicle length for subject i, with leg j, and in muscle region k.
- PA_{ijk} is the pennation angle for subject i, with leg j, and in muscle region k.
- MT_{ijk} is the muscle thickness for subject i, with leg j, and in muscle region k.
- MR_k is the muscle region k.
- H_i is the height of subject i.
- S_i is the sex of subject i.
- L_j is the leg j (e.g., dominant or non-dominant).
- u_i and v_i are the random effects for subject i in each model respectively.
- ϵ_{ijk} and η_{ijk} are the residual error terms for each model respectively.

R Code

```r
# Load necessary libraries
library(lme4)
library(mediation)

# Read the data file
meddata <- read.csv("path/to/your/meddata.csv")

# Mediator model
fit.mediator <- lmer(FL ~ MT * MR + H + S + L + (1|SUBJECTS), data=meddata)

# Outcome model
```

```
fit.outcome <- lmer(PA ~ MT * MR + FL * MR + H + S + L + (1|SUBJECTS), data=
    meddata)

# Conditional Direct, Indirect, and Total Effect Estimation (Table 2 and Table 3)
cdite.region.22 <- mediate(model.m = fit.mediator, model.y = fit.outcome, sims =
    1000, robustSE = F, treat = "MT", mediator = "FL", covariates = list(MR = 22))
cdite.region.39 <- mediate(model.m = fit.mediator, model.y = fit.outcome, sims =
    1000, robustSE = F, treat = "MT", mediator = "FL", covariates = list(MR = 39))

# Summary of conditional effects
summary(cdite.region.22)  ## Conditional effects for MR = 22%
summary(cdite.region.39)  ## Conditional effects for MR = 39%
```
